# Synthesis, Cytotoxic and Antimalarial Activities of Benzoyl Thiosemicarbazone Analogs of Isoquinoline and Related Compounds

**DOI:** 10.3390/molecules15020988

**Published:** 2010-02-23

**Authors:** Ratchanok Pingaew, Supaluk Prachayasittikul, Somsak Ruchirawat

**Affiliations:** 1 Department of Chemistry, Faculty of Science, Srinakharinwirot University, Sukhumvit 23, Bangkok 10110, Thailand; 2 Laboratory of Medicinal Chemistry, Chulabhorn Research Institute, Vipavadee-Rangsit Highway, Bangkok 10210, Thailand; 3 Program in Chemical Biology, Chulabhorn Graduate Institute and the Center of Excellence on Environmental Health, Toxicology and Management of Chemicals, Vipavadee-Rangsit Highway, Bangkok 10210, Thailand

**Keywords:** thiosemicarbazone, papaveraldine, cytotoxic activity, antimalarial activity

## Abstract

Thiosemicarbazone analogs of papaveraldine and related compounds **1–6** were synthesized and evaluated for cytotoxic and antimalarial activities. The cytotoxic activity was tested against HuCCA-1, HepG2, A549 and MOLT-3 human cancer cell lines. Thiosemicarbazones **1**–**5** displayed cytotoxicity toward all the tested cell lines, while compounds **2**–**5** selectively showed potent activity against the MOLT-3 cell lines. Significantly, N(4)-phenyl-2-benzoylpyridine thiosemicarbazone **4** exhibited the most potent activity against HuCCA-1, HepG2, A549 and MOLT-3 cell lines with IC_50_ values of 0.03, 4.75, 0.04 and 0.004 µg/mL, respectively. In addition, 2-benzoylpyridine thio-semicarbazones **3 **and **4 **showed antimalarial activity against *Plasmodium falciparum* with IC_50_ of 10^-7^ to < 10^-6^ M. The study demonstrates the quite promising activity of analog **4** as a lead molecule for further development.

## 1. Introduction

**Figure 1:**
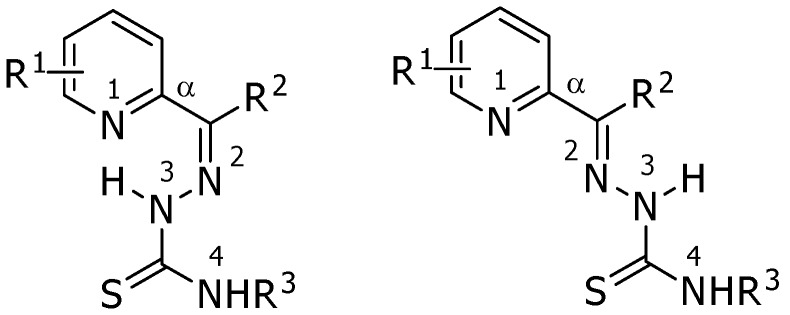
Strutures of α-(N)-heterocyclic thiosemicarbazones: R^1^-R^3^ = hydrogen, alkyl group or aryl group; Z (left) and E (right) geometrical isomers.

Thiosemicarbazones display a broad spectrum of pharmacological properties, including antitumor, antifungal, antibacterial, antiviral and antimalarial activities [1]. Much effort has been devoted to structural variations of the thiosemicarbazones for achieving the ultimate goal of medicinal applications [2,3,4,5,6,7]. The antitumor activity of such thio compounds was revealed in their ability to inhibit ribonucleotide reductase (RR), a necessary enzyme for DNA synthesis [8]. Currently, 3-aminopyridine-2-carboxaldehyde thiosemicarbazone, triapine, is being evaluated in human phase II trials as a cancer chemotherapeutic agent [9]. The thiosemicarbazone side chain located at a position α to the heterocyclic nitrogen, through a conjugated N-N-S tridentate ligand system, is essential for anticancer activity [4]. In general, two geometrical isomers about the imine double bond (*E* and *Z*) are possible for the thiosemicarbazones, of which the *Z* isomer is stabilized by an intramolecular hydrogen bond between N(3)-H and the heterocyclic nitrogen (Figure 1).

Activities of a number of α-(N)-heterocyclic carboxaldehyde thiosemicarbazones were reported [3,4,5,6,7]. However, isoquinoline-1-carboxaldehyde thiosemicarbazone (IQ-1), a 3,4-benzo derivative of pyridine-2-carboxaldehyde thiosemicarbazone (2-PT), was shown to be a significantly more potent inhibitor of RR than the parent compound and less toxic [4,5]. The result suggested that the occurrence of a hydrophobic interaction between the benzenoid moiety of the IQ-1 and the enzyme rendering the compound more active [5]. Although 1-formylisoquinoline thiosemicarbazone has been extensively investigated [6,7], studies of the 1-benzoyl analog have rarely been reported. This prompted us to explore the biological activities of thiosemicarbazone analogs of 1-benzoylisoquinoline. Papaveraldine, the most commonly known 1-benzoylisoquinoline, was used as a lead compound. The study presents the synthesis and an investigation of the cytotoxic and antimalarial activities of thiosemicarbazone analogs of this benzoylisoquinoline and related compounds.

## 2. Results and Discussion

### 2.1. Chemistry

**Figure 2:**
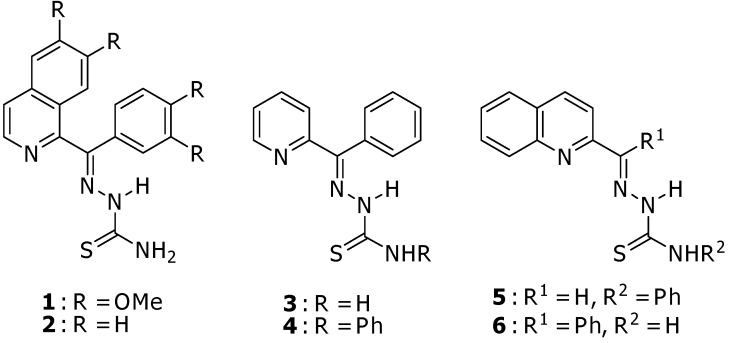
Thiosemicarbazone derivatives **1**–**6**.

Thiosemicarbazones **1–6** (Figure 2) were synthesized in good yields (75–88%) by mixing equimolar amounts of the corresponding carbonyl compounds with thiosemicarbazide derivatives in refluxing ethanol [10,11]. Thiosemicarbazones of 1-benzoylisoquinoline **2** and 2-benzoylquinoline **6** could be derived from 1-benzoylisoquinoline and 2-benzoylquinoline which were prepared from the Reissert compounds [12]. 

^1^H-NMR spectra of the benzoyl thiosemicarbazones **1**–**4 **and **6 **showed double signals as a consequence of the existence of a mixture of *Z* and E isomers. In comparison, the ^1^H- and ^13^C-NMR spectra of the known thiosemicarbazones **3** and **4** are in accordance with the previously reported data [13,14,15]. However, only the *E* isomer was observed for quinoline-2-carboxaldehyde thiosemicarbazone **5**. The preference for the *E* isomer may result from a significant difference in size between the quinoline ring and the hydrogen atom, which allows the steric effect between the quinoline ring and the thiosemicarbazone side chain to overcome the intramolecular hydrogen bonding. The stereochemistry of the thiosemicarbazone **5** was confirmed by a NOESY experiment which indicated a correlation between N(3)-H and N=C-H. 

### 2.2. Anticancer activity

The thiosemicarbazones **1**–**6** were tested for cytotoxic activity against HuCCA-1, HepG2, A549 and MOLT-3 human cancer cell lines (Table 1). It was obvious that the thiosemicarbazone analogs **1**–**5 **exerted cytotoxic activity in all the tested cell lines. Isoquinoline thiosemicarbazone **2** displayed about 30-fold higher inhibitory potency against MOLT-3 cells than papaveraldine thiosemicarbazone **1**, indicating that the presence of polar methoxy substituents decreased the cytotoxic effect. 2-Benzoyl-pyridine thiosemicarbazones **3** and **4** exhibited higher activity than the isoquinoline analog **2**. It is clear that the cytotoxic activity against MOLT-3 cells was remarkably improved by about 10-fold when the isoquinoline ring of **2** was replaced by a pyridine ring as in compound **3**. However, compounds **2** and **3 **displayed no significant effect on HuCCA-1, HepG2 and A549 cell lines. Apparently, the most active compound was N(4)-phenyl-2-benzoylpyridine thiosemicarbazone **4**, which showed inhibitory activity higher than doxorubicin against the HuCCA-1 and A549 cell lines. Moreover, a higher activity of **4** was observed against HepG2 and MOLT-3 cell lines when compared with etoposide. It is suggested that the N(4) side chain bearing a phenyl group distinctly enhances the cytotoxicity. Moreover, when we consider the benzo group connected either at the imine C-atom (as in **4**) or fused at the 5,6-position of the pyridine ring (as in **5**), the pyridine analog **4** remarkably exhibited much greater activity (IC_50_ of 0.004 µg/mL) than the quinoline **5** with an IC_50_ of 0.77 µg/mL. Therefore, this implies that the steric effect arising from the hydrophobic phenyl group at the imine carbon is important for potent cytotoxic activity. Unfortunately, cytotoxic activity of quinoline thiosemicarbazone **6** could not be determined owing to a solubility problem. However, it had been reported that the 2-benzoylpyridine thiosemicarbazones **3** and **4 **exhibited cytotoxic activity [11,13,16,17,18] against SK-N-MIC (neuroepithelioma), MCF-7 (breast cancer), TK-10 (renal carcinoma), UACC-62 (melanoma), K562 (leucocythemia) and BEL7402 (liver cancer) cell lines as well as antifungal activity [14,15] against *Candida albicans*. Moreover, the analog **4 **also displayed antimicrobial activity against *Staphylococcus aureus* [10].

**Table 1:** Cytotoxic activity^a^ of compounds **1**–**6** against four cancer cell lines.

Compound	Cytotoxic activity (IC_50_, µg/mL)			
	HuCCA-1	HepG2	A549	MOLT-3
**1**	36.0	31.5	30.0	25.83
**2**	35.0	16.0	30.0	0.72
**3** ****	25.0	17.5	24.0	0.088
**4** ****	0.03	4.75	0.04	0.004
**5**	5.5	11.75	3.6	0.77
**6^c^**	ND^b^	ND^b^	ND^b^	ND^b^
Etoposide^d^	ND^b^	16.0	ND^b^	0.024
Doxorubicin^e^	0.35	0.23	0.38	ND^b^

^a^ Cancer cell lines are: HuCCA-1 Human cholangiocarcinoma cancer cells; HepG2 Human hepatocellular liver carcinoma cell line; A549 Human lung carcinoma cell line; and MOLT-3 Human lymphoblastic leukemia cell line; ^b^ ND = Not determined; ^c^ insoluble in DMSO; ^d,e^ Etoposide and doxorubicin were used as the reference drugs.

### 2.3. Antimalarial activity

The study also tested antimalarial activity of the thiosemicarbazones **1**–**6** against *Plasmodiun falciparum* (Table 2). Results showed that the 2-benzoylpyridine thiosemicarbazones **3** and **4** exerted good antimalarial activity, with IC_50_ values of 10^-7^ to < 10^-6^ M, while the quinoline analog **6** displayed fair activity with an IC_50_ of 10^-6^ to < 10^-5^ M. Isoquinoline thiosemicarbazones **1** and **2** as well as quinoline thiosemicarbazone **5** were shown to be inactive antimalarials with IC_50_ > 10^-5^ M. 

**Table 2:** Antimalarial activity of compounds **1**–**6** against *P. Falciparum.*

Compound^a^	activity	IC_50_ (M)
**1–2, 5**	Inactive	> 10^-5^
**3–4**	Good	10^-7^ to < 10^-6^
**6**	Fair	10^-6^ to < 10^-5^

^a^ Chloroquine hydrochloride was used as the reference drug.

## 3. Experimental

### 3.1. General

Column chromatography was performed using silica gel 60 (70–230 mesh ASTM). Analytical thin-layer chromatography (TLC) was performed with silica gel 60 F_254_ aluminium sheets. ^1^H- and ^13^C- NMR spectra were recorded on a Bruker AVANCE 300 NMR spectrometer (operating at 300 MHz for ^1^H and 75 MHz for ^13^C). FTIR were obtained using a universal attenuated total reflectance attached on Perkin–Elmer Spectrum One spectrometer. Mass spectra were recorded on a Finnigan INCOS 50 and Bruker Daltonics (microTOF). Melting points were determined using a Griffin melting point apparatus and are uncorrected. 

### 3.2. Synthesis of thiosemicarbazones **1–6**

Glacial acetic acid or conc. hydrochloric acid were added to a mixture of an appropriate carbonyl compound and thiosemicarbazide or N(4)-phenylthiosemicarbazide in absolute ethanol and the resulting mixture was then heated under reflux for 6–48 h (completion of reaction was monitored by TLC). The reaction mixture was cooled, and the separated crystalline solid was filtered off, wash with ether and recrystallized from ethanol.

*Papaveraldine thiosemicarbazone* **(1)**. Papaveraldine [19] (707 mg, 2 mmol), thiosemicarbazide (182 mg, 2 mmol), EtOH 20 mL and conc. HCl (5 drops) yielded **1** (639 mg, 75%). mp (EtOH) 200–202 ºC literature [20] 230 ºC; IR ν_max_ 3324, 3288, 3128, 1629, 1609, 1509, 837, 777, 734 cm^-1^;^ 1^H-NMR (DMSO-d_6_) δ 3.73, 3.86, 4.06 (3s, 12H, 4 × OCH_3_), 6.32 (dd, *J* = 8.4, 1.7 Hz, ArH), 6.80 (d, *J =* 8.6 Hz, Ar*H*), 6.83 (s, ArH), 7.11–7.30 (m, ArH), 7.68 (d, *J =* 1.6 Hz, ArH), 7.86 (s, ArH), 7.90 (s, ArH), 8.35 (d, *J =* 6.2 Hz, ArH), 8.64 (d, *J =* 6.1 Hz, 1H, ArH), 8.45, 8.63, 9.22, 9.25, 10.68 (5s, 3H, N3-H and N4-H_2_); ^13^C-NMR (DMSO-d_6_) δ 55.3 (OCH_3_), 55.9 (OCH_3_), 56.0 (OCH_3_), 56.3 (OCH_3_), 56.4 (OCH_3_), 56.5 (OCH_3_), 57.2 (OCH_3_), 57.4 (OCH_3_), 104.0, 107.0, 108.2, 108.8, 109.7, 111.5, 113.2, 118.2, 122.2, 122.5, 122.9, 123.9, 125.8, 128.6, 130.7, 132.8, 134.6, 135.2, 137.4, 140.0, 145.7, 148.3, 148.6, 149.6, 150.1, 150.4, 151.1, 151.4, 152.9, 156.7, 179.5 (C=S); LRMS (EI)*m*/*z* (%) 426 (26) [M]^+^, 393 (40), 367 (37), 366 (100), 352 (35), 351 (43), 339 (31), 338 (96), 337 (47), 324 (36), 322 (46), 321 (39), 306 (36), 204 (34), 189 (31); HRMS (TOF) *m*/*z* [M+H]^+^ calcd for C_21_H_23_N_4_O_4_S 427.1435 found 427.1441.

*1-Benzoylisoquinoline thiosemicarbazone* (**2**). 1-Benzoylisoquinoline (1.166 g, 5 mmol), thiosemicarbazide (456 mg, 5 mmol), EtOH 30 mL and conc. HCl (5 drops) gave **2** (1.209 g, 79%). m.p. (EtOH): 150–152 ºC; IR ν_max_ 3423, 3327, 3256, 3155, 3057, 1584, 1558, 1470, 1385, 1258, 1114, 936, 875, 823, 691 cm^-1^;^ 1^H-NMR (DMSO-d_6_) δ 7.25–7.75 (m, ArH), 7.83 (d, *J =* 8.7 Hz, ArH), 7.93 (t, *J* = 7.1 Hz, ArH), 8.10–8.28 (m, ArH), 8.34 (d, *J* = 8.2 Hz, ArH), 8.85 (d, *J* = 5.8 Hz, ArH), 8.42, 8.59, 9.10, 9.13, 10.09 (5s, 3H, N3-H and N4-H_2_);^ 13^C-NMR (DMSO-d_6_) δ 122.4, 123.1, 125.2, 126.5, 127.1, 127.3, 127.8, 128.5, 128.6, 128.7, 129.0, 129.2, 129.8, 129.9, 130.2, 130.3, 131.3, 134.5, 136.1, 136.2, 136.5, 137.7, 142.9, 145.8, 148.6, 148.8, 152.8, 178.9 (C=S); LRMS (EI)*m*/*z* (%) 306 (65) [M]^+^, 289 (20), 246 (26), 218 (77), 217 (100), 216 (38); HRMS (TOF) *m*/*z* [M+H]^+^ calcd for C_17_H_15_N_4_S 307.1012 found 307.1008.

*2-Benzoylpyridine thiosemicarbazone* (**3**). 2-Benzoylpyridine (916 mg, 5 mmol), thiosemicarbazide (456 mg, 5 mmol), EtOH 30 mL and glac. CH_3_COOH (5 drops) furnished **3** (1.126 g, 88%). m.p. (EtOH) 151–153 ºC; IR ν_max_ 3418, 3345, 3256, 3149, 3055, 1607, 1459, 852, 791, 704 cm^-1^;^ 1^H-NMR (DMSO-d_6_) δ 7.25–7.66, 8.49 (m and d, *J* = 8.0 Hz, 12H, ArH), 7.85, 8.00 (2td, *J* = 8.0, 1.3 Hz, 1H, C4-ArH), 8.42, 8.84 (2d, *J* = 4.6 Hz, 1H, C6-ArH), 8.16, 8.58 (2s, 2H, N4-H_2_), 8.73, 12.54 (2s, 1H, N3-*H*);^ 13^C-NMR (DMSO-d_6_) δ 121.9, 124.2, 125.1, 126.2, 128.5, 128.7, 129.0, 129.4, 129.6, 131.1, 136.7, 136.9, 138.4, 143.8, 148.7, 149.1, 149.2, 151.4, 154.5, 178.1 (C=S), 178.6 (C=S); LRMS (EI)*m*/*z* (%) 257 (19) [M+H]^+^, 256 (78) [M]^+^, 239 (12), 196 (30), 195 (17), 178 (12), 168 (81), 167 (100), 166 (16); HRMS (TOF) *m*/*z* [M+H]^+^ calcd for C_13_H_13_N_4_S 257.0855 found 257.0853.

*2-Benzoylpyridine N(4)-phenyl thiosemicarbazone* (**4**). 2-Benzoylpyridine (916 mg, 5 mmol), N(4)-phenylthiosemicarbazide (836 mg, 5 mmol), EtOH 30 mL and glac. CH_3_COOH (5 drops) gave **4** (1.345 g, 81%). m.p. (EtOH) 138–139 ºC literature [10] 135 ºC; IR ν_max_ 3305, 3056, 1594, 1513, 798, 693 cm^-1^;^ 1^H-NMR (DMSO-d_6_) δ 7.17–7.71, 8.42 (m and d, *J* = 7.9 Hz, 12H, ArH), 7.86, 8.01 (2td, *J* = 7.9, 1.5 Hz, 1H, C4-ArH), 8.46, 8.84 (2d, *J* = 4.4 Hz, 1H, C6-ArH), 8.93, 10.28 (2s, 1H, N4-*H*), 10.67, 13.08 (2s, 1H, N3-H);^ 13^C-NMR (DMSO-d_6_) δ 122.6, 124.5, 125.3, 125.9, 126.1, 126.4, 128.5, 128.6, 128.8, 129.3, 129.6, 129.9, 130.7, 131.2, 136.8, 137.0, 138.5, 138.9, 144.3, 149.0, 149.1, 149.7, 151.5, 154.3, 176.5 (C=S), 176.7 (C=S); LRMS (EI) *m*/*z* (%) 333 (18) [M+H]^+^, 332 (100) [M]^+^, 254 (22), 239 (27), 212 (21), 201 (25), 196 (23), 168 (58), 167 (39), 93 (49), 51 (23); HRMS (TOF) *m*/*z* [M+H]^+^ calcd for C_19_H_17_N_4_S 333.1168 found 333.1169.

*2-Formylquinoline N(4)-phenyl thiosemicarbazone* (**5**). 2-Formylquinoline (786 mg, 5 mmol), N(4)-phenylthiosemicarbazide (836 mg, 5 mmol), EtOH 30 mL and conc. HCl (5 drops) furnished **5** (1.178 g, 77%). m.p. (EtOH) 168–170 °C literature [21] 184 ºC; IR ν_max_ 3314, 3109, 1592, 1539, 824, 756, 689 cm^-1^;^ 1^H-NMR (DMSO-d_6_) δ 7.23 (t, *J* = 7.5 Hz, 1H, C4'-ArH), 7.39 (t, *J* = 7.5 Hz, 2H, C3'-ArH_2_), 7.55 (d, *J* = 7.5 Hz, 2H, C2'-ArH_2_), 7.61 (t, *J* = 8.0 Hz, 1H, C6-ArH), 7.77 (t, *J* = 8.0 Hz, 1H, C7-ArH), 7.98 (d, *J* = 8.0 Hz, 1H, C5-ArH), 8.02 (d, *J* = 8.0 Hz, 1H, C8-ArH), 8.33 (s, 1H, CN-H), 8.37 (d, *J* = 8.7 Hz, 1H, C4-ArH), 8.60 (d, *J* = 8.7 Hz, 1H, C3-ArH), 10.39 (s, 1H, N4-H), 12.19 (s, 1H, N3-H);^ 13^C-NMR (DMSO-d_6_) δ 118.6 (C3), 125.8 (C4'), 126.4 (C2'), 127.3 (C6), 128.0 (C4a, C5), 128.2 (C3'), 128.8 (C8), 130.1 (C7), 136.4 (C4), 139.0 (C1'), 143.1 (C=N), 147.4 (C8a), 153.8 (C2), 176.6 (C=S); LRMS (EI) *m*/*z* (%) 306 (100) [M]^+^, 245 (46), 186 (83), 175 (90), 170 (51), 142 (60), 115 (95); HRMS (TOF) *m*/*z* [M+H]^+^ calcd for C_17_H_15_N_4_S 307.1012 found 307.1004.

*2-Benzoylquinoline thiosemicarbazone* (**6**). 1-Benzoylisoquinoline (1.166 g, 5 mmol), thio-semicarbazide (456 mg, 5 mmol), EtOH 30 mL and conc. HCl (5 drops) gave **6** (1.255 g, 82%). m.p. (EtOH): 210–212 ºC; IR ν_max_ 3468, 3349, 3042, 1570, 767, 702 cm^-1^;^ 1^H-NMR (Pyridine-d_5_) δ 7.25–7.97 (m, 9H, Ar*H*), 8.13, 8.20 (2d, *J* = 8.7 Hz, 1H, Ar*H*), 8.29, 8.48 (2d, *J* = 8.4 Hz, 1H, ArH), 9.10, 9.36, 9.89, 10.21 (4s, 2H, N4-H_2_), 10.38, 14.75 (2s, 1H, N3-H); ^13^C-NMR (Pyridine-d_5_) δ 119.8, 123.3, 124.0, 127.6, 127.8, 128.1, 128.2, 128.4, 128.7, 128.9, 129.5, 129.6, 129.7, 130.0, 130.2, 131.2, 136.0, 136.4, 137.8, 138.2, 142.9, 146.6, 148.0, 149.2, 150.3, 152.7, 181.3 (C=S); LRMS (EI) *m*/*z* (%) 306 (68) [M]^+^, 246 (33), 245 (39), 218 (100), 217 (78); HRMS (TOF) *m*/*z* [M+H]^+^ calcd for C_17_H_15_N_4_S 307.1012 found 307.1008.

### 3.3. Cytotoxic activity assay

The cytotoxic assay was performed as previously described by Tengchaisri and co-workers [22]. Briefly, cell lines suspended in RPMI 1640 containing 10% FBS were seeded at 1 × 10^4^ cells (100 µL) per well in 96-well plate, and incubated in humidified atmosphere, 95% air, 5% CO_2_ at 37 ºC. After 24 h, additional medium (100 µL) containing the test compound and vehicle was added to a final concentration of 50 µg/mL, 0.2% DMSO, and further incubated for 3 days. After that, the cells were fixed with EtOH–H_2_O (95:5, v/v), stained with crystal violet solution, and lysed with a solution of 0.1 N HCl in MeOH, after which absorbance was measured at 550 nm. The number of surviving cells was determined from the absorbance. Etoposide and doxorubicin were used as the reference drugs (Table 1).

### 3.4. Antimalarial activity assay

Antimalarial activity of the tested compounds was evaluated against chloroquine-resistant (T9/94) *P. falciparum* using the literature method [23]. Before performing the experiment, *P. falciparum* culture was synchronized by using sorbitol-induced hemolysis according to the method of Lambros and Vanderberg [24] to obtain only ring-infected cells and then incubated for 48 h prior to the drug testing to avoid effect of sorbitol. The experiments were started with synchronized suspension of 0.5% to 1% infected red blood cell during ring stage. Parasites were suspended with culture medium supplemented with 15% human serum to obtain 10% cell suspension. The parasite suspension was put into 96-well microculture plate; 50 µL in each well and then add 50 µL of various tested drug concentrations. These parasite suspensions were incubated for 48 h in the atmosphere of 5% CO_2_ at 37 ºC. The percentage of parasitemia of control and drug-treated groups were examined by microscopic technique using methanol-fixed Giemsa stained thin smear blood preparation. The efficacy of the drugs was evaluated by determining the drug concentration that reduced parasite growth by 50%. 

## 4. Conclusions

The thiosemicarbazone analogs of isoquinolines **1**–**2 **and quinoline **5**, including the benzoylpyridine thiosemicarbazones **3** and **4**, possessed moderate to excellent cytotoxic activity against the HuCCA-1, HepG2, A549 and MOLT-3 human cancer cell lines. Although the benzo group fused to the heterocyclic ring decreased the cytotoxic activity, conversely, the addition of a phenyl group on the N(4) side chain and at the imine carbon significantly enhanced the potency of the compounds. It is concluded that the hydrophobic group is responsible for the biological activities. Additionally, benzoylpyridine thiosemicarbazones **3** and **4**, as well as quinoline analog **6**, showed mild to good antimalarial activity. The finding suggests that some of these compounds might serve as potential candidates for anticancer and antimalarial agents, particularly, the analog **4** is quite a promising lead compound.
